# Ulcer metastasis? Anatomical locations of recurrence for patients in diabetic foot remission

**DOI:** 10.1186/s13047-020-0369-3

**Published:** 2020-01-13

**Authors:** Brian J. Petersen, Gary M. Rothenberg, Priti J. Lakhani, Min Zhou, David R. Linders, Jonathan D. Bloom, Katherine A. Wood, David G. Armstrong

**Affiliations:** 1 Podimetrics Inc, Somerville, MA USA; 2 University of Michigan Medical School, Ann Arbor, MI USA; 3 Einstein Healthcare Network, Philadelphia, PA USA; 4 Keck School of Medicine, University of Southern California, California, Los Angeles USA

**Keywords:** Diabetic foot, Diabetic foot ulcer, Recurrence, Prevention, Epidemiology

## Abstract

**Background:**

The “cancer analogy” is powerful for communicating risk to and organizing care for patients with diabetic foot syndrome. One potentially underappreciated similarity between cancer and foot ulcers is that both can recur at anatomical locations distinct from the primary occurrence, albeit with different physiological mechanisms. Few studies have characterized the location of diabetic foot ulcer recurrence, and these have been limited by considering only the first recurrent wound following a recent-healed wound. We therefore characterized the anatomical locations at which diabetic foot ulcers are likely to recur considering multiple wounds during follow-up and the locations of all prior wounds documented in the participant’s history.

**Methods:**

We completed a secondary analysis of existing data from a 129 participant multi-center study of participants in diabetic foot remission. The primary outcome was plantar foot ulceration, and each participant was followed for 34 weeks or until withdrawing consent, allowing characterization of all wounds occurring. We stratified the anatomical locations of wounds prior to the trial by the following outcome categories during the trial: no recurrence, recurrence to the same anatomical location, recurrence to a different anatomical location on the same foot, and recurrence to the contralateral foot.

**Results:**

A large percentage (48%) of wounds recurred to the contralateral foot, and the proportion of subsequent foot ulcer to the contralateral limb was largely unaffected by the anatomical location of foot ulcer prior to the study. Only 17% of prior diabetic foot ulcers were followed by recurrence to the same anatomical location. Rates of recurrence remained high during treatment of a wound (0.41 foot ulcer/ulcer-year). Participants had documented wounds to 2.2 distinct anatomical locations on average, and more than 60% of participants had wounds to more than one plantar location by the end of the study.

**Conclusions:**

Given the significant morbidity, mortality, and resource utilization associated with foot ulcer recidivism, quality and evidenced-based preventive care is essential. Our results better characterize the burden of recurrence and to what anatomy recurrence is most likely. These insights may benefit providers and patients alike for the provision of high-quality preventive care thereby resulting in reduced morbidity, mortality, and cost.

**Trial registration:**

The study providing the data for this secondary analysis was registered on ClinicalTrials.gov (NCT02647346) on January 6, 2016. The study was retrospectively registered.

## Background

Since Armstrong and colleagues highlighted that the five year mortality rates associated with foot ulceration and lower extremity amputation exceed several common cancers [1], including those of the breast and prostate, the “cancer analogy” has become an effective education and communication tool for practitioners and patients alike. The analogy has helped convey the seriousness of diabetic foot syndrome and has helped guide conversations regarding expectations and outcomes. Consider a diabetic foot ulcer (DFU) as a “malignancy” of the foot, and “wound hospice” as a viable alternative to conventional treatment. Diabetic foot “remission” is a key concept for helping patients and caregivers appreciate the ongoing risk of recurrence after a wound has healed.

The cancer analogy is appropriate: both cancer and DFU are associated with significant mortality [2] and reduced quality of life [3]; both have substantial burden of illness, confronting patients and caregivers alike with time-consuming and challenging treatment regimens [4]; both are commonly refractory and chronic [5]; both are associated with recidivism [6]; and both result in considerable resource utilization and cost [7].

One potentially underappreciated similarity between DFU and cancer is that both can recur at anatomical locations distinct from the primary occurrence, albeit with different underlying physiological mechanisms. Few studies have characterized the location of DFU recidivism relative to the location of previous wounds [8–11]. These studies found that only a minority of wounds (between 19 and 37%) recur at the same anatomical location as a patient’s most recently-healed DFU. In fact, a 2017 paper by Ornheholm and colleagues [8] reported that 38% of recurrent plantar forefoot DFU (excluding minor digits) are to the contralateral limb.

The risk of recurrence to the contralateral limb is additionally reflected in research reporting rates of lower extremity amputations (LEA). In the year following a LEA, approximately 10% of patients have a second amputation of the contralateral limb [12–16], and as many as 50% have amputation of the contralateral limb within five years following a primary amputation [13, 17–19]. While LEA rates overall have improved over recent decades [20], this survey of the literature suggests that secondary LEA rates to the contralateral limb have not meaningfully improved over several generations.

Unfortunately, the existing literature on the location of DFU recurrence is limited in two important ways. First, these studies considered only the first recurrence and did not report on all locations of previous DFU. Second, existing data were not stratified granularly by anatomical location. We therefore report on a secondary analysis of existing data in order to better characterize the anatomical locations at which DFU have recurred in study participants. These data may inform improved preventive practice, leading to better patient outcomes.

## Methods

We abstracted data from a multicenter trial in 129 participants (NCT02647346), which assessed the accuracy of a remote temperature monitoring floor mat (Podimetrics RTM System; Somerville, MA) [21]. This trial was conducted across seven outpatient sites in the United States. Participants were required to have a history of DFU, be ambulatory, and have documented ankle-brachial index exceeding 0.5 in the absence of palpable pedal pulses. Patients with unhealed DFU, active Charcot foot disease, end-stage renal disease, or immunosuppressive disease were not eligible for participation. The study was conducted in accordance with the Helsinki Declaration and was approved by multiple institutional review boards as required by the individual enrollment centers.

Each participant was followed under standard diabetic foot care for 34 weeks or until withdrawing consent. Upon enrollment, investigators conducted a chart review to characterize the anatomical locations of previous DFU for each participant. During the study, participants were instructed to contact the investigators if a lesion (defined as an anatomical area which has suffered injury or damage) or DFU was noted during daily self-exam. All lesions and wounds were treated consistent with standard practice. Additionally, each participant was contacted by phone during months 2 and 4 of the trial and asked if he or she noticed any change to either foot. At the conclusion of the trial, each participant was evaluated in clinic for any lesions and wounds, and study staff completed a final chart review to confirm that all relevant DFU were captured as study outcomes.

The primary outcome during the trial was recurrence of plantar DFU with non-acute etiology. We adopt the definitions of the International Working Group on the Diabetic Foot [22] and define a “foot ulcer” as a break of the skin of the foot penetrating through the epidermis and at a minimum through part of the dermis. “Healed” was defined as macroscopic epithelialization. We define a “recurrent foot ulcer” as a foot ulcer developing after healing of a previous foot ulcer, located at any site on either foot. Participants were followed through the end of the trial or until withdrawing consent, thus allowing investigators to characterize incidence of all plantar DFU occurring during participation. During the trial, 37 participants had a total of 53 recurrent plantar DFU (0.63 DFU/participant-year) to all limbs.

We stratified DFU outcomes during the trial into the following categories:

1. the participant remained in diabetic foot remission, i.e. had no recurrent foot ulcers during the trial
2. the participant had a recurrent foot ulcer to the same anatomical location as a prior wound
3. the participant had a recurrent foot ulcer to a different anatomical location on the same foot as a prior wound
4. the participant had recurrent foot ulcer to the contralateral foot

Additionally, we calculated the number of distinct locations of DFU (considering each digit, metatarsal head, arch, and heel as a distinct location) occurring to each participant through the end of the trial, including those documented in the patient’s medical records prior to study enrollment.

## Results

In this secondary analysis of existing data, we reported on the breakdown of the anatomical locations of DFU both prior to and during the trial. During the 34 week trial, 92 participants (71.3%) remained in diabetic foot remission. In those who did have a recurrent foot ulcer during the trial (28.7%), 48% of DFU recurred to the contralateral foot, 35% of DFU recurred to a different anatomical location on the same foot, and 17% recurred at the same location as a previous DFU.

Table 1 shows the distribution of DFU locations on a per-participant basis. Prior to the trial, participants were most likely to have DFU history at the hallux (57%), lesser digits (34% combined to the third, fourth, and fifth toes), second digit (28%), 1st metatarsal head (26%), and heel (7%). During participation, the most common locations for recurrence were 1st metatarsal head (41%), hallux (32%), and 5th metatarsal head (16%).

**Table 1:** Participants with Foot Ulcers Stratified by Anatomical Location Prior to and during the Study

Anatomical Location	Percentage of Participants with DFU at Location	
	Prior to Study	During Study
Hallux	56.6%	32.4%
2nd Digit	27.9%	8.1%
1st Metatarsal Head	25.6%	40.5%
3rd Digit	13.2%	10.8%
5th Metatarsal Head	12.4%	16.2%
3rd Metatarsal Head	12.4%	8.1%
5th Digit	11.6%	5.4%
2nd Metatarsal Head	11.6%	8.1%
4th Digit	8.5%	0.0%
4th Metatarsal Head	7.0%	5.4%
Heel	7.0%	8.1%

**Fig. 1:**
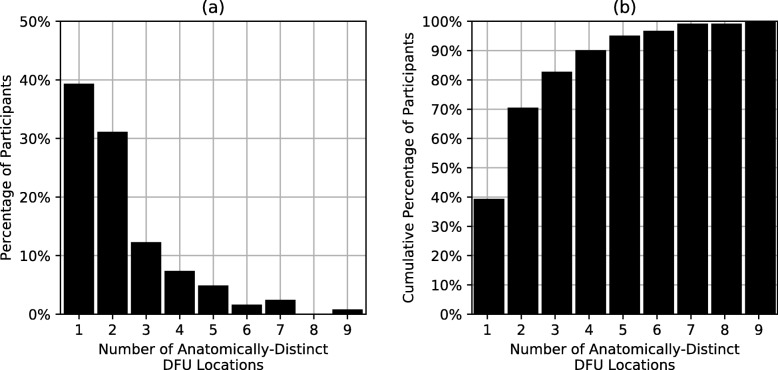
(**a**) the discrete distribution of number of distinct anatomical locations of foot ulcers; (**b**) the cumulative distribution of number of distinct anatomical locations of foot ulcers. The cumulative distribution (**b**) shows the percentage of participants with wounds to fewer than or equal to *N* anatomical locations, where *N* is the x-axis value

Figure 1 shows the distribution of anatomically-distinct DFU locations for participants through the end of the study. Upon study completion, participants had a history of DFU to 2.2 +/− 2.0 distinct anatomical locations on average. More than 60% of participants had DFU to more than one plantar location, and one participant had history of DFU to nine distinct anatomical locations.

**Fig. 2:**
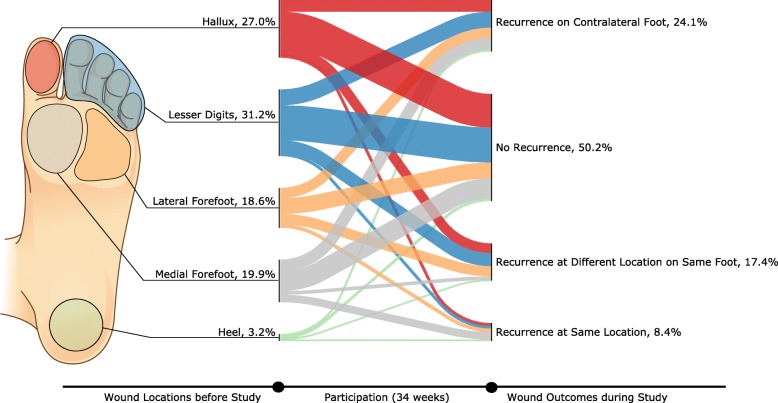
The anatomical locations of foot ulcers occurring prior to the study and the outcomes during participation

**Fig. 3:**
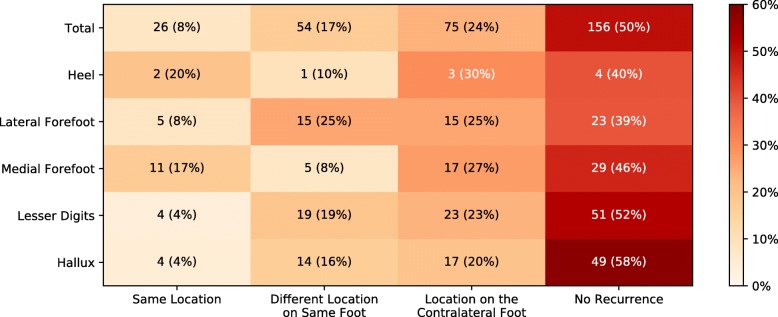
The anatomical locations of foot ulcers occurring prior to the study and the outcomes during participation

Whereas Table 1 shows the percentage of participants with wounds to different locations, Figs. 2 and 3 show the anatomical locations of wounds prior to the trial and subsequent outcomes during the trial as a percentage of total number of wounds documented. Medial forefoot DFU were disproportionately more likely to recur at the same location (17.7%) than other anatomical locations, while lateral forefoot DFU were most likely to be followed by a DFU at another location on the same foot (25.9%). Approximately half of recurrent DFU in cases where the previous DFU was to the first metatarsal head were followed by contralateral recurrence (52%, 17/33). Similarly, nearly half (49%, 17/35) of previous hallux DFU were followed by contralateral recurrence during the trial. The proportion of subsequent DFU to the contralateral limb was largely unaffected by the anatomical location of DFU prior to the study.

A total of four foot ulcer recurrences occurred to two participants being treated for an unhealed DFU that occurred during the trial (0.41 DFU/ulcer-year). Two of these recurred to the contralateral limb during treatment (0.20 DFU/ulcer-year).

## Discussion

Our results suggest that DFU to patients in diabetic foot remission frequently occur in anatomically-distinct locations, including a large percentage to the contralateral foot. We reported a larger proportion of recurrence to the contralateral foot than Orneholm and colleagues [8], with 48% of the DFU incident during the 34 week trial recurring to the limb opposite a prior DFU. Only 17% of prior DFU were followed by recurrence to the same anatomical location during follow up. Additionally, patients undergoing treatment for unhealed wounds maintain high recurrence rates despite increased clinical attention during treatment: we report a high rate of incidence to participants being treated for an unhealed DFU that occurred during the trial (0.41 DFU/ulcer-year).

These data have important implications for preventive care. Major risk factors for DFU, which include peripheral neuropathy and peripheral arterial disease, commonly affect the entirety of both limbs. Given this fact and our results, preventive care should be provisioned for both feet and not only on areas of previous ulceration or concern. Accordingly, patients and caregivers should be educated on the elevated risk to both feet and instructed to thoroughly examine the entirety of both feet daily for discoloration, callus, blisters, fissures, and other pre-ulcerative findings.

Given our findings of high incidence to the foot without a wound during treatment for a DFU, providers should also be cognizant of the risk to both limbs and let this insight guide practice. For example, while treating a DFU, providers should remain attentive to foot without a wound, which may be predisposed to elevated risk not only chronically due to neuropathy and arterial disease but also acutely due to gait deviation and pressure redistribution secondary to treatment of the wounded foot.

Additionally, our results may be helpful for interpreting the data from emerging telemedicine technologies for the diabetic foot, such as once-daily foot temperature monitoring [23]. These new monitoring modalities have been shown to improve outcomes for those in diabetic foot remission [24–26], allowing for early and targeted intervention much like mammography and colonoscopy for breast and colon cancer, respectively. A better understanding of patient risk, including the anatomical locations of recurrence, can provide additional context for those utilizing such emerging modalities for the diabetic foot in remission.

Unlike previous investigations, one benefit of our current effort is that we considered the anatomical locations of all DFU documented in each participant’s history, not just the most recent. This design has allowed us to fully characterize the burden of recurrence for those patients with diabetic foot syndrome, which is sobering. A majority of participants (60%) had a history of DFU at two distinct anatomical locations prior to the trial, and nearly one in five participants had history of DFU to four or more plantar locations on the feet. Prior to the trial, more than half of participants (57%) had a history of DFU to the hallux, and more than a quarter had a previous DFU to the 1st metatarsal head. During the trial, recurrence was less likely to the digits, possibly as a result of prior amputation of distal anatomy, which was highly prevalent among the participants [21].

## Conclusion

In our data, DFU to participants in diabetic foot remission frequently occurred to anatomy different from the locations of previously-healed wounds. A large percentage (48%) of wounds recurred to the contralateral foot, and only 17% of prior DFU were followed by recurrence to the same anatomical location. Rates of recurrence remained high during treatment (0.41 DFU/ulcer-year). Overall participants had documented wounds to 2.2 distinct anatomical locations on average, and more than 60% of participants had DFU to more than one plantar location.

Given the significant morbidity, mortality, and resource utilization associated with DFU recidivism, quality and evidenced-based preventive care is essential. Our results better characterize the burden of recurrence and also may inform improved understanding of the anatomical locations of recurrence. These insights may improve practice of preventive care for those in diabetic foot remission, thereby reducing morbidity, mortality, and cost.

## Data Availability

All data generated or analyzed during this study are included in this published article.

## Abbreviations

DFU: Diabetic foot ulcer
LEA: Lower extremity amputation
